# Editorial

**DOI:** 10.1002/osp4.100

**Published:** 2016-12-28

**Authors:** David B. Sarwer

I wanted to end 2016 with a brief update on the status of *Obesity Science & Practice*. As the publishing team has been targeting, we were able to publish a fourth issue of the journal in 2016, which accompanies this editorial. We are thrilled with this accomplishment, as is Wiley, the publisher of the journal. Reaching our fourth issue in the calendar year for a new, open access journal, and this early in the history of the journal, is a strong sign of our early growth ‐ not to mention the potential of the journal. This issue again contains a nice mix of studies of children and adults, coming from authorship teams from throughout the world. Over 40% of our submissions are international. I am very pleased with this mix.

Perhaps the best news of all, we are well on our way to having a sufficient number of papers accepted for publication for our first issue of 2017. Some of these papers have been directly referred to *Obesity Science & Practice* from the other Wiley obesity journals‐‐*Obesity, Obesity Reviews, Pediatric Obesity, and Clinical Obesity.* We are seeing an increase in the number of referred articles and I am grateful for the support from the editorial teams from those journals, as well as the leadership teams from The Obesity Society and World Obesity, for their work to support our early success.

I would also like to take this opportunity to highlight some of the high quality research we have published in 2015 and 2016. We continue to see readership metrics grow and are pleased to announce the most downloaded articles in 2016:

- Fast food, soft drink and candy intake is unrelated to body mass index for 95% of American adults
  Just, D. and Wansink, B
- Adherence to low‐carbohydrate and low‐fat diets in relation to weight loss and cardiovascular risk factors
  Hu, T., Yao, L., Reynolds, K. et al.
- Proxy measures of vitamin D status – season and latitude – correlate with adverse outcomes after bariatric surgery in the Nationwide Inpatient Sample, 2001–2010: a retrospective cohort study
  Petersen, L. A., Canner, J. K. Cheskin, L. J. et al.

The article by Just and Wansink was downloaded more than 2000 times, while the article by Hu and his colleagues received more than 1800 downloads in 2016. All three of these papers received mass media attention and were circulated via Twitter, blogs and other electronic media mechanisms. The article by Petersen and his colleagues was mentioned in several news media outlets. We anticipate that several of the papers recently published in the journal will follow this trajectory and I am pleased to see the growing interest from our readers.

In 2016, we also welcomed Ms. Lena Jacobsen from Wiley to the team. Lena serves as the new Journals Publishing Manager and we will work closely together to continue to keep the journal on its positive trajectory, and work on new initiatives for the journal. As *Obesity Science & Practice* is in the early stages of index evaluation with Thomson Reuters, continuing to publish a steady stream of high quality, impactful articles is our key objective for the new year.

In 2017, I am looking forward to the continued growth of *Obesity Science and Practice*. I thank you for your continued support and hope that you will consider *Obesity Science & Practice* for your future publications. I wish you and yours nothing but the best for the holidays and new year.

